# A Comparison of Computational Models for Eukaryotic Cell Shape and Motility

**DOI:** 10.1371/journal.pcbi.1002793

**Published:** 2012-12-27

**Authors:** William R. Holmes, Leah Edelstein-Keshet

**Affiliations:** 1 Department of Mathematics, University of British Columbia, Vancouver, British Columbia, Canada; 2 Department of Mathematics, University of California, Irvine, California, United States of America; Indiana University, United States of America

## Abstract

Eukaryotic cell motility involves complex interactions of signalling molecules, cytoskeleton, cell membrane, and mechanics interacting in space and time. Collectively, these components are used by the cell to interpret and respond to external stimuli, leading to polarization, protrusion, adhesion formation, and myosin-facilitated retraction. When these processes are choreographed correctly, shape change and motility results. A wealth of experimental data have identified numerous molecular constituents involved in these processes, but the complexity of their interactions and spatial organization make this a challenging problem to understand. This has motivated theoretical and computational approaches with simplified caricatures of cell structure and behaviour, each aiming to gain better understanding of certain kinds of cells and/or repertoire of behaviour. Reaction–diffusion (RD) equations as well as equations of viscoelastic flows have been used to describe the motility machinery. In this review, we describe some of the recent computational models for cell motility, concentrating on simulations of cell shape changes (mainly in two but also three dimensions). The problem is challenging not only due to the difficulty of abstracting and simplifying biological complexity but also because computing RD or fluid flow equations in deforming regions, known as a “free-boundary” problem, is an extremely challenging problem in applied mathematics. Here we describe the distinct approaches, comparing their strengths and weaknesses, and the kinds of biological questions that they have been able to address.

## Introduction

From the earliest embryogenesis, through growth and development, cells in our body undergo programmed rearrangements and relative motion that shapes tissues, generates the form of the organism, and maintains its integrity despite constant environmental pressures. How cells move is thus an intriguing problem in biology, not only in the context of metazoans but also in far simpler single-celled organisms such as amoebae. Modern biology and advanced imaging techniques have allowed an increasingly fine inspection of the molecular processes underlying the complex process of cell locomotion. But as with many other biological investigations, making sense of the voluminous data is a challenging undertaking.

Partly for this reason, there has been increased impetus to complement experimental observations with theoretical treatment of the problem of cell movement, with the idea of breaking down the very intricate mechanisms into simplified prototypes that can be understood more readily. This review summarizes some of the recent approaches that have addressed single cell motility from a theoretical and computational perspective. Here we focus primarily (but not exclusively) on single eukaroytic cells that undergo chemotaxis or directed motion, rather than, for example, epithelia or cell clusters.

Many motile eukaryotic cells described here have a thin sheet-like front edge, the lamellipod, known to be the major determinant of cell shape and motility. Devoid of organelles and filled with the cytoskeletal protein actin (polymerized into filaments, F-actin), it is the protrusion motor that extends the cell forward. Retraction of the rear along with choreographed formation, maturation, and breakage of cell-substrate adhesions complete the motility machinery. Front extension and rear retraction are generally observed to be orthogonal to the edge of the cell. Some cells are constantly deforming, while others achieve a relatively stable steady-state shape as they crawl (reviewed below). In the latter case, this mandates that there be a graded distribution of extension and retraction (“graded radial extension,” GRE) [1] so as to preserve the shape and size of the cell as it moves.

Cells of distinct types differ in certain respects, but all eukaryotes contain F-actin and major signalling proteins such as small GTPases, phosphoinositide-3-kinase (PI3K), phosphatase and tensin homolog (PTEN), and other regulatory molecules that impinge on the cytoskeleton. Fluorescence imaging, speckle microscopy, total internal reflection fluorescence (TIRF), and confocal and electron microscopy have revealed the structure of the cytoskeleton, the spatial redistribution of actin, its nucleators (e.g., Arp2/3), and its regulators, as well as localization dynamics of single molecules in ever-increasing detail. In principle, data are plentiful and should allow for an accurate understanding of the machinery of cell motion. In practice, the presence of complex molecular interactions, crosstalk, and feedback make it very challenging to decipher underlying mechanisms and how they are coordinated.

Here we survey the types of theoretical efforts that have been devoted to gaining insight into basic aspects of cell motility. As we will see, most of these efforts include some consideration of (1) cytoskeletal dynamics or (2) regulatory signalling. Many models link that biochemistry to mechanical forces and material properties (e.g., viscoelasticity) of the cell material. Each aspect on its own is already a challenging theoretical problem. The difficulties associated with the second are lack of detailed knowledge about the molecular interactions in signalling networks. The challenge in the first is the issue of how to describe the cell material (elastic, fluid, or viscoelastic). Confounding the problem even more is the fact that biochemistry and biophysics of the cell are intimately connected to changes in its shape and movement. This means that the combined biochemistry/biophysics needs to be represented in a continually deforming 2-D or 3-D domain in what is known as a “free boundary problem” in applied mathematics. This significantly raises the bar for entry into this exciting area of investigation.

**Figure 1:**
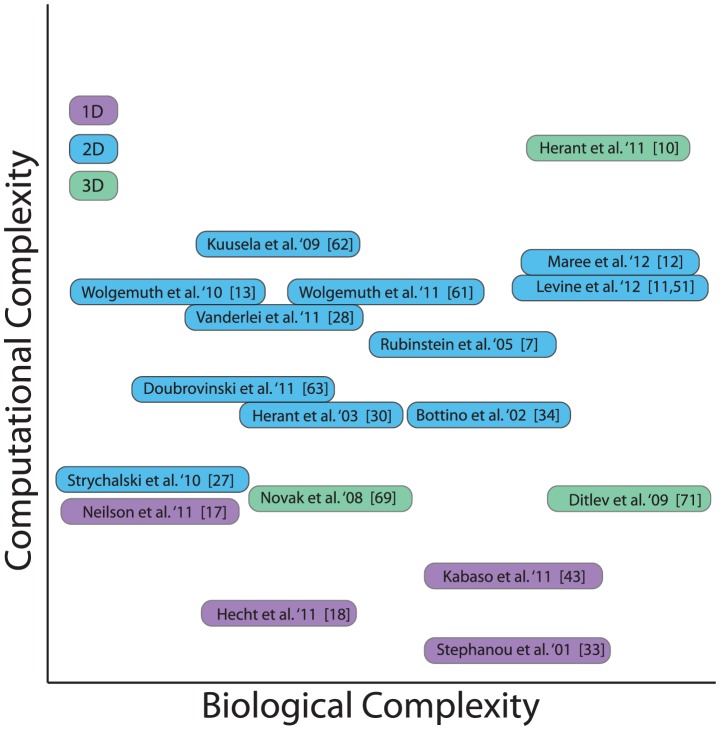
Summary of model complexity. A summary of the models on a two-axes “complexity” plane. The horizontal axis represents the level of biological detail (increasing from left to right) and the vertical axis represents the computational complexity of the simulation (increasing from bottom to top). See Table 1 for a listing of specific biological and computational aspects of these models.

In the sections that follow, we first survey some of the specific properties of eukaryotic cells, favorite experimental subjects that are also used as modeling prototypes. We discuss both universal features of such cells and distinctions that affect how they are viewed theoretically. The kinds of scientific questions driving the computational research are often cell-type specific, and especially so in recent articles that closely link models and experiments. We then summarize some of the recent work in which 2-D or 3-D motility is simulated and discuss both the biological and computational complexities that must be addressed, outlined in Figure 1 and Table 1. Particular attention is paid to how free boundary computations have been circumvented, or solved, and how the cell material has been represented in the wide variety of simulations currently in use. We conclude with some perspectives and open challenges for the future.

**Table 1:** Summary of published motility models.

	Model Reference	Model Components					Computational Methods	
		CD	CF	SD	MM	AD	Boundary	PDE
1D	Neilson 2011 [17]			x	x		LSM	FEM
	Hecht 2011 [18]			x	x		LMP	FDM
	Kabaso 2011 [43]	x			x	x	LMP	FDM
	Stephanou 2008 [33]	x			x	x	LMP	FDM
2D	Bottino 2002 [34]	x					LMP	FEM
	Herant 2003 [30]	x	x				LMP	FEM
	Doubrovinski 2011 [64]	x		x	x		LMP	FDM
	Rubinstein 2005 [7]	x				x	LMP	FEM
	Rubinstein 2009 [9]	x				x	N/A	FEM
	Wolgemuth 2010, 2011[13], [48]		x	x		x	LSM	FVM
	Levine 2012 [11], [52]	x		x	x	x	PFM	FDM
	Vanderlei 2011 [28]		x	x	x		LMP	FDM
	Marée 2006, 2012 [8], [12]	x		x	x	x	CPM	FDM
	Kuusela 2009 [63]	x	x			x	LSM	FDM, FVM, FEM
3D	Novak 2008 [70]	x					N/A	FVM
	Ditlev 2009 [72]	x					N/A	FVM
	Herant 2010 [10]	x	x			x	LMP	FEM

Model components and computational methods of articles surveyed in this review. In columns 2–6 an “x” indicates inclusion (to some extent) of a given layer of biological complexity. Column heading abbreviations are as follows: CD, cytoskeleton dynamics; CF, cytosolic flow; SD, signaling dynamics; MM, membrane mechanics; AD, adhesion dynamics. The boundary treatment column (column heading Boundary) indicates the method used to handle the moving boundary of the cell: LMP, LSM, PFM, or the CPM. The PDE Methods column (column heading PDEM) indicates the procedure used to discretize model PDEs: Finite Difference Method (FDM), Finite Volume Method (FVM), or Finite Element Method (FEM).

## Universal Principles and Cell-Specific Phenomena

The goals of modelling and simulation efforts are not merely to reproduce observed motility properties (though that in itself is a challenge). Rather, it is to use the computational platforms to pose and address a variety of specific questions about cell motility and to understand the relationship between functional aspects of cell machinery and resulting form.

The variety of motile eukaryotic cells leads to two complementary issues: (1) finding universal principles that are conserved across species and (2) determining what is specific to a given cell type and how to explain such unique features. A recent review article [2] summarized major similarities and differences of cell types in the context of polarization behaviour. Here we describe motility differences and common features, together with some of the major issues that are addressed by modellers.

### Cell Types and Cell-Specific Modelling Questions

#### Budding yeast

The budding yeast, *Saccharomyces cerevisiae*, exhibits polarization in mating and in the genesis of daughter cells by budding. Mating yeast grow into schmoo-like shapes in response to mating pheromone gradients. The formation of buds is associated with localized buildup of the GTPase Cdc42 in a polar cap. Such systems have been used to motivate signalling models [3], [4], with a variety of 1-D, 2-D, and 3-D computations. Here we do not review the extensive literature on yeast as these cells are inherently nonmotile.

#### Fish keratocyte

Fish scale keratocytes have been a favourite cell for motility studies, as they have a fairly stable shape and persistent motion, with a broad flat front edge, and overall “canoe” or “D” shape. The relative simplicity of motion of this type of cell has attracted many models and simulations [5]–[12]. Keratocyte motility has been described by the GRE scheme, leading naturally to the question of whether GRE can be explained based on the underlying cytoskeletal dynamics [6] and how retraction, adhesion, and protrusion are coordinated to maintain the shape of the cell [7]. (For example, how are the side edges prevented from spreading outwards or retracting inwards? How does retraction at the rear keep up with protrusion at the front? [13]) Keratocytes are among the fastest motile cells, with typical speeds of 10–40 µm/min. Many works have aimed at deciphering the connections between the actin recycling and acto-myosin dynamics and the speed of the cells [6], [7], [14].

#### Social amoeba

The Social amoeba *Dictyostelium discoideum* is another popular organism for study, with known genetics and ease of genetic manipulation. Models for the amoeboid motion of *Dictyostelium* include [15]–[19]. These rapid chemotactic cells respond to the chemoattractant cAMP and undergo random motion in its absence. In contrast to stable keratocyte motion, cell shape continually deforms, with frequent and random sprouting of pseudopods. One recurrent question of interest to modellers has been what mechanism accounts for the continual generation of these protrusions [15] and how they vie for dominance to lead to the observed motility phenotype [17], [18]. Researchers have also questioned whether signalling activity patches are a requirement for chemotaxis or whether pseudopod mechanics suffices to account for it [17]. Biologically, it has been found that the pseudopod that senses highest cAMP levels usually becomes the dominant one [20], allowing cells to easily change direction in response to changing cues. Due to ease of mutant and genetic manipulation, the PI3K-PTEN and phosphoinositide (PI) pathways have been well-studied in these organisms. One of the earliest works showed that these proteins and lipids polarize rapidly in response to cAMP gradients, even when the actin cytoskeleton is disrupted by drugs such as latrunculin. These findings engendered many models for the signalling and gradient-sensing aspects of these cells, primarily in 1-D computations [21]–[24].

#### Neutrophils

Neutrophils, a component of the white blood cells, are rapid responders to pathogen-associated stimuli. They are expert chemotactic cells, with crawling speeds up to 10–20 µm/min and persistent motility once stimulated. The role of the small GTPases (Cdc42, Rac, Rho) in polarization and signalling to actin had been investigated fairly early experimentally [25], [26] and led to a number of recent modeling studies. These addressed a number of questions, including how internal signalling systems interface with the deforming cell shape [8], [12], [27], [28], how they lead to robust polarization [8], [29], and how cells with such internal signalling can remain sensitive to new stimuli [8].

Neutrophil shape is more fluid than that of keratocytes, with a fairly symmetric resting state and a highly polarized shape when stimulated. In the absence of pathogen stimuli, neutrophils “roll” along the endothelial lining of blood vessels, but they change shape and invade the tissues by extravasation when encountering inflammatory or pathogenic signals. Neutrophils are also well-known phagocytes. The actin cytoskeleton plays a vital role in both motility and phagocytosis. Models addressing neutrophil behaviour have been aimed at understanding how these cells modulate their cortical tension to organize motility and phagocytosis [30], and how details of the cytoskeletal mechanics (e.g., network swelling, mechanical repulsion, contractile forces, etc.) can be reconciled with the expanding local surface area [30]–[32].

#### Fibroblasts

Fibroblasts are cells of the connective tissue responsible for wound healing. By comparison with the previously described cells, their motion is sluggish, with speeds of only 1 µm/min. The shapes of these cells are largely dictated by their strong adhesion to the substrate, mediated by large complexes called focal adhesions. A question investigated by [33] is how hydrostatic pressure, actin-mediated contraction, and focal adhesion formation relate to the traction forces exerted by the cell. A recent 3-D model that investigates the genesis of a typical fibroblast shape is [10].

#### Nematode sperm

The nematode sperm is a crawling cell, with a flat lamellipodium, a dome-like cell body, and an elongated shape in the direction of crawling. This cell has a simplified cytoskeleton consisting of the major sperm protein (MSP) whose assembly and disassembly appears to be influenced by an internal pH gradient. While detailed biology of this cell and biopolymer are still vague, these have been the subject of several modeling articles [34]–[36]. One of the earliest 2-D computational articles on cell motility [34] addressed the issue of how MSP contributes to motility. The effect of MSP properties (e.g., stiffness and anisotropy) and of the cell size and shape on crawling speed were investigated in [36].

### Overarching Modelling Goals

From the above list, it is clear that motile cells include both fast and slow, persistent moving and static resting phenotypes, with either stable shapes, or frequent and randomly distributed protrusive activity. Hence, in considering models for cell motility, it is clear that goals and questions posed have been, to some extent, specific to cell type. At the same time, a few questions of more universal applicability recur as themes in some of the modeling articles. These include some of the following: (1) What is the relationship between actin dynamics, cell shape, and movement [7]? (2) How does the acto-myosin network self-organize? What accounts for the distribution of myosin and the flow of actin observed in crawling cells [9]? (3) How do distinct processes such as myosin contraction, transport-limited G-actin polymerization into F-actin, vesicle delivery by microtubules (MTs), and GTPase signalling contribute to cell shape generation [13]? (4) How can cells make decisions when multiple conflicting cues are presented to them? How can cells navigate in complex environments [12], [19]? (5) What accounts for morphology differences between cell types [10], [37]?

Roughly speaking, the theoretical models can be classified into several types. (1) Some models are concerned exclusively with biophysical aspects of the actin cytoskeleton in varying degrees of detail. These are aimed at understanding how distribution of actin and its effectors such as myosin and adhesion complexes affect the shape and locomotion speed of cells. (2) Other models have a greater focus on signalling and chemotactic orientation. Often the details of the cytoskeleton are omitted and signalling activity is implicitly linked to forces causing protrusion and retraction. (3) A few efforts combine aspects of both these approaches. (4) In some cases, phenomenological or rule-based computations are used to generate cell shape, using a highly abstracted representation of the cell.

## Cell-Shape Models in One Spatial Dimension (1-D)

Here we survey several influential models that describe “cell shape” effectively in a single spatial dimension. Some models depict only the leading edge, while others are perimeter models that represent the cell as a closed curve (that is, a periodic 1-D domain). That boundary curve is then identified with the cell “membrane,” and the 2-D interior of the shape is identified as the “cytosol,” generally assumed to be spatially uniform. Two major model categories are noteworthy. In the first, particular attention is paid to steady cell motion and the role of mechanical forces on cell shape and locomotion speed. These models pertain primarily to keratocytes (i.e., to cells whose shapes are preserved as they move). They incorporate various biophysical elements such as actin growth, adhesion dynamics, membrane curvature, and tension. A second model category pertains to the more dynamic amoeboid motion, typical of *Dictyostelium*. Most such models eschew cytoskeleton dynamics and consider mainly the dynamics of signalling in an attempt to understand the feedbacks responsible for the formation and interaction of pseudopods. Whereas in category I, the goal of modelling is to understand how individual biophysical attributes contribute to steady-state cell shape and motility, in class II, the goal is to investigate how signalling and pseudopod dynamics contribute to chemotaxis.

### Biophysics of Steady Motion

#### Keratocyte motion

**Figure 2:**
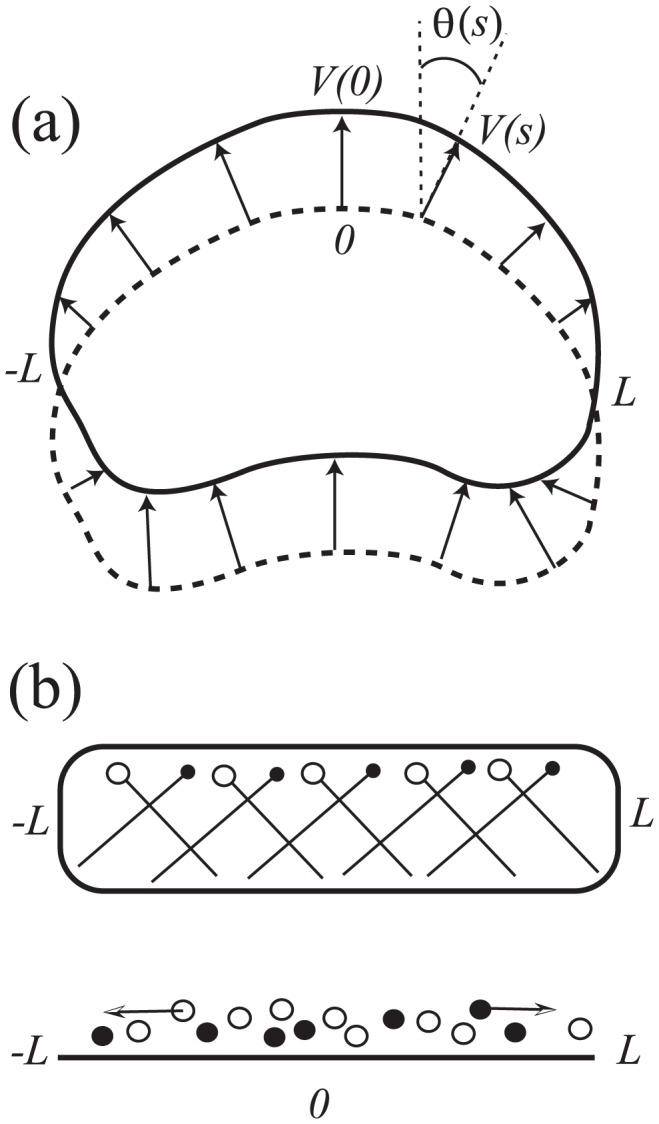
Graded Radial Extension Model. (a) To preserve the shape of the cell as it moves, forward extension has to be graded along the front (rear) edges of the cell. In the GRE model [1], the velocity at a point on the cell edge is perpendicular to the edge and of magnitude *V*(*s*) = *V*(0)cos(*θ*(*s*)), where *s* is arclength along the cell front edge (−*L*≤*s*≤*L*) and *V*(0) is the translocation velocity of the cell. (b) In Grimm et al. [6], only actin filament barbed ends at the leading edge are modeled. These are approximated by two populations, representing ends moving right (black dots) and those moving left (white dots). Arp2/3-mediated branching of one type gives rise to the other type. The cell edge is approximated as nearly flat (nearly rectangular shaped cell), and the evolving density of the actin filament tips is modeled in 1-D using a continuum (partial differential) equation.

The fact that the shape of a keratocyte is intimately linked to the F-actin distribution across its lamellipod is well-recognized [38], [39]. As previously noted, protrusion of a keratocyte perimeter satisfies a GRE property [1]. Let *s* be arc-length distance along the cell's front edge with −*L*≤*s*≤*L* spanning that edge from left to right. Then GRE states that the protrusion rate (orthogonal to the edge) at *s* satisfies *V*(*s*) = *V*(0)cos(*θ*(*s*)), where *θ*(*s*) is the angle between the edge normal at *s* and the direction of motion, as shown in Figure 2a. This has led to the obvious question of how this is achieved via cytoskeletal growth at the leading edge. A subset of the class of models for the leading edge of keratocytes are aimed at understanding F-actin distribution, force velocity relationships, and their role in the GRE and steady motion. These models consider only the leading edge of such cells, with a domain −*L*≤*x*≤*L* where 2*L* is the cell diameter.

In a pioneering article, Grimm et al. [6] proposed a simple 1-D model for short, straight actin filaments growing at ±35° along a relatively flat leading edge. The edge shape is described by a function *y* = *f*(*x*). Projected along a single (*x*) axis, the two filament density classes (*ρ* ^+^(*x*,*t*), *ρ* ^−^(*x*,*t*)), black and white tips, respectively, in Figure 2b) appear to move laterally with velocity ±*v*. Filaments in a given class produce daughters in the other class, an approximation of the Arp2/3 branching nucleation. Filaments also appear to decay as they are capped and prevented from keeping up with the steady motion of the cell edge (in the “*y*” direction). The resulting model, a pair of partial differential equations of hyperbolic type, is elegant in its simplicity, allowing for explicit solutions in several limiting cases. The effects of capping and local versus global competition for Arp2/3 could then be carefully dissected. Predicted actin profiles are symmetric, with a peak at the center of the front edge. The local-Arp2/3 model compares well with experimental observations for keratocyte cell-edge actin distributions. The GRE results in a relationship between the protrusion velocity *V_n_*(*x*) and slope of the edge at *x*, (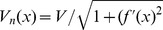). The authors used this, together with experimental actin density, to arrive at an approximate relationship between protrusion velocity and local total actin density, *V_n_*(*x*)≈*V*_0_ exp(−ω/(*ρ*(*x*)−*ρ*_0_)). The model is fit to experimental data, and best fits correspond to relatively low capping rate. In this case, the filaments persist longer and average out local density fluctuations better. Thus, one of the insights gained by the model is that relatively low capping is consistent with shape stability, whereas a high rate of capping is not.

The model of [6] later engendered more detailed experiment-theory rounds such as [40]. There, the role of the actin-associated protein Ena/VASP was investigated experimentally and theoretically to test its proposed role as a capping competitor/inhibitor. Equations of [6] were accordingly modified to track barbed ends associated with VASP (hence protected from capping). The model was used to establish that a high level of VASP at edge relative to interior leads to a greater population of pushing barbed ends, and thus lower membrane resistance per actin filament, and faster protrusion. Experimentally, the authors identified both canoe-shaped, “coherent” cells, with a smooth edge, and stable persistent motion and decoherent D-shaped cells (with ruffled edge and wobbly motion). The former were observed to have higher VASP level at the edge. The model predicts that at the range of lower resistance per filament, protrusion is limited by G-actin (barbed ends compete for resources), rather than F-actin, whereas resistance limits protrusion at the sides of the cell when the F-actin density is lower. All in all, this article is an example of the adoption of a theoretical model as a tool in experimental research, and it provides support for the anti-capping role of VASP.

In fact, several other works that will be described more fully as 2-D investigations were also linked to 1-D reductions, used to gain valuable insights about the role of the cytoskeleton in motility. Among these, we mention the simulated nematode sperm and keratocyte motility (reviewed further on) [7], [34] as notable examples. The elastic and gel swelling forces due to MSP were explored in [34] to analytically establish the stress distribution across the cell. In [7], a 1-D reduction was used to obtain the distribution of myosin and contractile forces at the rear of the cell, again using analytic solutions to reduced 1-D partial differential equations (PDEs). Such reductions only treat simplified limiting cases or special systems where notable tricks can be used to transform and solve the system (as in [6]). But they provide additional insights for how various parameters (capping, nucleation rates, binding, or unbinding rates) influence qualitative behaviour in the models.

So far, we have surveyed 1-D models that represent part or all of the cell perimeter, as these blend well into a discussion of cell shape. Another large class of 1-D models focuses on a transect of the cell from the edge inwards (parallel to the direction of motion). Such models are less suited for describing cell shape and are primarily focused on addressing how actin, myosin, and/or other cell constituents are distributed away from the cell edge. An example includes [14], reviewed later, and others too numerous to list here. The two examples [41], [42] emphasize continuum mechanics. The former treats a 1-D cell as a viscoelastic material with active stresses and interaction with the substrate to compute cell speed in a fibroblast cell type. The latter similarly considers actomyosin as a treadmilling viscoelastic 1-D strip where contraction is assumed to be proportional to myosin density. The F-actin density determines the elastic, viscous, and adhesion properties of the cell. One result obtained is that lower adhesion strength and gel elasticity, together with stronger contractility, merely shortens the “cell” but does not strongly affect its speed. Such early models have simple geometry and simple constitutive relations, and so lead at best to qualitative, rather than quantitative, agreement with observed cell behaviour.

#### Perimeter models with membrane curvature and adhesion

Another subset of models describing steady motion are less concerned with the GRE mechanism associated with keratocytes and concentrate more on the role of membrane effects and adhesion dynamics. These consider a 1-D periodic domain representing cell perimeter. The work by [43] presents a 1-D model for the periphery of a 2-D cell based on the idea that proteins (with BAR domains) interact with membrane curvature to localize and direct actin growth, edge protrusion, and adhesion. They show that feedback between the distribution of convex proteins and protrusive activity leads to symmetry breaking instabilities (analyzed by linear stability analysis) and probe the respective roles of actin protrusion strength versus adhesion strength on cell shape.

In this relatively complex 1-D model, both curvature and tension effects are incorporated in a Helfrich free energy functional. Curvature-associated proteins are assumed to decrease local membrane tension by promoting adhesion formation and/or by increasing local intrinsic curvature. (This leads to positive feedback as proteins induce curvature and are at the same time drawn to regions of high curvature.) The authors consider two limiting cases of protrusion versus adhesion strength. In the case of strong adhesion activity with no actin activity, cells take on an elongated shape with a single high curvature peak. In the opposite regime (high actin activity and no adhesion), the result is a broadly distributed flat front with two high curvature peaks.

Another work that explores the role of adhesion is that of [33]. This 1-D periphery model describes the interplay of hydrostatic pressure, actin-mediated contraction, focal adhesion formation, and traction forces in fibroblast motility. Three adhesion states are considered: spots, focal complexes, and focal adhesions. These differ in lifetime, with only the latter capable of producing traction forces for motility. It is assumed that stresses lead to the maturation of focal adhesions and that focal adhesions themselves result in larger stresses, creating positive feedback. Model results are compared to experiments reported in the same article, with cell speed and area being compared. One finding is that cell speed is maximal at some intermediate adhesion strength, in agreement with experiments. Another is that cell speed is directly related to adhesion lifetime and inversely related to recycling time.

### Signalling and Dynamic Cell Motility

The above class of models was largely aimed at understanding steady cell motion. In this section, we consider a second class of 1-D models for amoeboid motion that treat a cell as a 1-D periodic domain representing the cell edge. The motion of amoeboid cells is much less regular than that of keratocytes. For example, *Dictyostelium* chemotaxis is driven by the formation, retraction, and splitting of pseudopods. Hence models of amoeboid motion have a greater focus on explaining transient signalling activity on the membrane, on production and competition of pseudopods, and on the role of pseudopod dynamics in chemotaxis, rather than internal cytoskeletal function or steady motility.

Recall that in *Dictyostelium* cells, the response to chemoattractant gradients is a rapid formation of patches of activity of proteins (PI3K, Ras) and lipids (PIP_3_) at the edge corresponding to the gradient direction. This is evident even in cells whose cytoskeleton is frozen by drugs such as latrunculin. The same components also concentrate at the leading tips of pseudopods during cell motion. Hence, a central concern in many such models is what sets up and accounts for the dynamics of such activity patches, a problem of chemical pattern formation on the 1-D cell perimeter. Because the details of the molecular interactions and the roles of distinct players are still controversial, many models are based on hypothetical pattern-formation systems. Underlying such models are reaction diffusion (RD) equations for the concentrations of two or more reactants (activator *u*, inhibitor *v*) of the form:
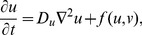
(1)

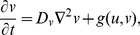
(2)where *D_u_*<*D_v_* (necessary for pattern formation) and *f*,*g* are nonlinear interactions that lead to some type of spontaneous pattern formation. In some cases, the inhibitor is viewed as global (*D_v_*→∞). In these models, the perimeter is parameterized by 0<*x*≤*c*, where *c* is the cell circumference and periodic boundary conditions *u*(0,*t*) = *u*(*c*,*t*), *v*(0,*t*) = *v*(*c*,*t*) are imposed. In this view, only the edge of the cell is considered, and no dorsal/ventral membrane or cytoplasm is included. (Later 2-D models will implicitly account for these structures.) Below, we describe examples of this type of 1-D model.

#### Static perimeter models

The idea that intracellular chemical signalling patterns can dictate the polarization and orientation of cells can be found in early articles by Gierer and Meinhardt such as [44], [45]. Here, the concept of lateral inhibition was used to show that local activation together with long-ranged inhibition can produce activity peaks of chemicals. The locations of those peaks could be biased externally by a stimulus, represented, for example, by a random input in the RD system. The interacting activator-inhibitor system is then interpreted as a cellular signalling system governing the polarization response of cells (reviewed in [2]).

As the activator-inhibitor systems described in early articles tend to lock into a fixed pattern, modifications were made to account for the sensitivity of a cell to new stimuli. Among such work, a more recent highlight is the article of Meinhardt [15] where a local inhibitor (to unfreeze pattern) was added to the previous local activator, and global inhibitor. In [15], the activity of reacting chemicals is concentrated at the circumference of a static disk, with a uniformly distributed global inhibitor. The solutions to the set of RD equations are taken to indicate the nascent sites of pseudopod extension. Of specific interest in this work is the exploratory formation of pseudopods in cells such as *Dictyostelium discoideum* and in neuronal growth cones. An external gradient biases the RD system, promoting a patch of activator that polarizes the cell. Multiple filopodia can be generated if the spatial range of the inhibitor is smaller than the cell perimeter, whereas a single “front” occurs when the inhibitor is global. The retraction of old filopods and creation of new ones mandates the presence of a third species that acts as a long time scale local inhibitor. In [15], gradient sensing by oscillating RD systems, competition of multiple sites for filopodia, and generation of traveling waves along the cell perimeter were shown as proof of principle for the capacity of such systems to mimic cell behaviours. This article was a precursor to 2-D motility modelling. It sparked much interest and led to a resurgence of research on the orientation and direction-sensing of chemotactic cells.

Recent work of a similar flavour but distinct in the details includes the models for coupled local excitation global inhibition (LEGI, see [21]) modules describing PI3K, its downstream effector, the PI PIP_3_, and the phosphatase tensin homology protein (PTEN) on the perimeter of *Dictyostelium* [22]. These models are geared at understanding direction sensing and adaptation during chemotaxis and are not concerned with patterns of spontaneous activity or pseudopod generation. Some of the models compared well with experiments on immobilized *Dictyostelium* amoebae responding to cAMP [23].

#### Deforming cell edge perimeter models

More recently, similar ideas about activator–inhibitor interactions on the perimeter of a domain have advanced to track a deforming cell edge. A RD system capable of producing chemical patterns is postulated as before to account for cell signalling. The boundary curve is discretized, the motion of its nodes, and hence the deformation of cell shape, is then related to the chemical activity pattern by linking either forces or velocities to the active signal concentrations. In some cases, additional representations of the cell edge mechanics are included. The models then investigate the feedback between the chemical patterning and cell shape. The models are concerned with the formation, retraction, and splitting of activated regions (pseudopods), and most are focused on *Dictyostelium* as the model organism. One technical issue that arises with these models is the need to remesh the boundary due to spreading or compression of nodes.

One example of perimeter modelling with deformable cell edge was provided by [46], which used a so-called “shape machine” to link the deformation of the domain to activity on its perimeter. This technique for computing the deforming boundary is based on spokes radiating outwards from a center of mass whose position is updated as the cell deforms. The rules for membrane deformation do not take into account forces, tension due to perimeter stretching, nor conservation of area (or volume) of the cell, which means that the motility mechanism is somewhat abstract. Nevertheless, this model fits into a class of models where membrane protrusion is directly linked to the signalling activity. The rule-based computation of [46] proceeds from a local-stimulation-global-inhibition system, with positive feedback in the protrusion signals and retraction signals that track some overall average (integral) of the protrusion signals. The local protrusive signals obey a (discretized) RD equation with stochastic input. The model consists of some 10 variables with 11 free parameters. The parameter space is wide enough to allow for a variety of motility phenotypes that resemble evolving shapes of real cells (fibroblasts, keratocytes, and neuronal growth cones).

In the above models for *Dictyostelium*, the signalling system is invoked to act as an internal compass, and all other processes are assumed to be downstream “readouts” of this information processing. However, Insall and Mackenzie [16], [17] model intrinsic cyclic growth of pseudopods and assume that external gradients and the internal signalling bias some steps in that cycle (not merely the initiation stage). They adopt a system of local activator, local inhibitor, and global inhibitor, similar to [15]. Their additional equation for the signal that induces activity is related to local receptor occupancy, in contrast to [15]. In these simulations, the cell perimeter moves out in a direction normal to the cell edge, with local speed proportional to the level of the activator. A phenomenological equation for “cortical tension” that tracks the changes in cell area was used to compute the velocity of retraction. A challenge is to track the expanding edge on which reaction and diffusion take place, and this is handled by remeshing and by implementing an equation that results in area preservation. A goal of this study was to test previously proposed models hypothesizing an intrinsic pseudopod cycle and whether these can predict chemotaxis behaviour in *Dictyostelium*. The results compare experimental and theoretical cell shapes and motility behaviour in response to manipulated gradients. These include initial polarization to a graded cue, persistent locomotion, as well as reorientation to new applied stimuli. A novel prediction of this work is that the direction of motion relative to the applied gradient will influence the angle at which new pseudopods split off from preexisting ones. This was found to agree well with experiments of real cells.

In a similar treatment of the same target organism, *Dictyostelium discoideum*, Hecht et al. [18] implemented a two-component excitable RD model (with an activator satisfying cubic kinetics and a first-order kinetics inhibitor) on a deforming 1-D periodic membrane (see also [24] for proposed excitable system governing cell navigation). This system is meant to describe stochastic, transient patches of activity governing membrane protrusions. The activator was meant to mimic RasGTP, observed experimentally to spatially colocalize with membrane protrusions. A separate model component leads to the determination of the cell front and rear based on external attractant field and internal “compass” with superimposed noise. Here the RD system determines only the location of activity patches that form pseudopods, not the direction-sensing of the cell. The force on the membrane depends on activator levels, cortical tension, and membrane curvature. For example, an expression of the form:
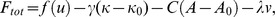
was used to represent the force orthogonal to the edge. Here *u* is the local activator concentration, *κ* is local membrane curvature in 2-D, *A* the area of the cell, and *λ* is a drag coefficient. Varying the excitability parameter across the cell perimeter then provides for cells that have a more sensitive (excitable) front. Results were compared with cells that were vertically restricted so that fluorescent membrane patches could be imaged readily. Inclusion of a noise term in the RD equations leads to spontaneous splitting of pseudopods, with no additional splitting mechanism required, and the model could reproduce observed cell shapes and chemotaxis behaviour.

The models [17], [18] address an issue that has been somewhat controversial in *Dictyostelium* motility, namely whether chemotaxis of these cells necessitates the formation of highly localized patches of special signalling agents such as PI3K, Ras, or their effectors, regulated by some signalling circuits. Localized patches are seen, and [18] explains how these could give rise to chemotaxis, but experimental literature has dealt several blows to claims that these are “essential.” The model in [17] demonstrates that chemotaxis may be possible without signalling proteins or signal processing separate from pseudopods.

As in many models for cell motility, special problems arise when multiple spatial cues are presented to the model cell. In [18], the original model is unable to account for winner-take-all pseudopod tug of war, so the authors adopted a second module that includes the availability of a “limited resource” *G*(*t*) for protrusion. The same type of model was also used by Hecht et al. [19] to describe cells moving through more complex environments, such as mazes. The authors showed that cells that secrete a repulsive chemical are better able to navigate through mazes. Both issues have also been addressed in a recent 2-D model [12], reviewed further on.

All in all, most models with 1-D domains either target the cytoskeleton and its distribution across the front edge in keratocytes, or the signalling and evolution of dynamic patterns along the cell perimeter, but not both. As we shall see, more complex models that consider rheological aspects, cytoskeleton, and/or signalling pathways have been developed in the context of 2-D simulations, as described in the next section.

## Simulations in Two Spatial Dimensions (2-D)

Computationally, we can group the 2-D models into classes of increasing complexity. Some are essentially simulations with irregular but fixed cell shapes. Those that do allow for deformation and evolution of the cell shape do so by one of several techniques. In principle, two separate aspects require special treatment. First, a method of choice has to keep track of the moving boundary. And second, some technique must be used to solve the interior problem, usually (but not always) a set of RD transport equations. We review specific computational methods below.

### Moving Boundary Formulation

The single most difficult aspect of motility modelling is the treatment of a moving deformable domain. There are four basic techniques for handling the boundary description and motion in these problems: (1) Lagrangian marker method, (2) level sets, (3) phase fields, and (4) Cellular Potts Model (CPM), alternatively known as the Glazier-Graner-Hogeweg Model (GGH) after the innovators who first adapted its use to biology. Each of these has its strengths and weaknesses. We briefly describe these before discussing specific models. In Table 1, we also indicate the method used in each of the models under review.

#### Lagrangian marker point (LMP)

The most natural method of dealing with a moving deformable domain is to define the boundary by a set of LMPs that are advected with some velocity inherent to the problem. The strengths of this method are its intuitive simplicity and the ability to compute local tension when the edge is deformed. This can be an important aspect of force balance at the cell edge. The downside of this treatment is the difficulty associated with application of boundary conditions and problems that arise when domains become either highly curved or stretched in a local region, requiring remeshing of the boundary. An example of this type of method is the immersed boundary method, where a fluid velocity field advects material marker points on the boundary.

#### Level set method (LSM)

**Figure 3:**
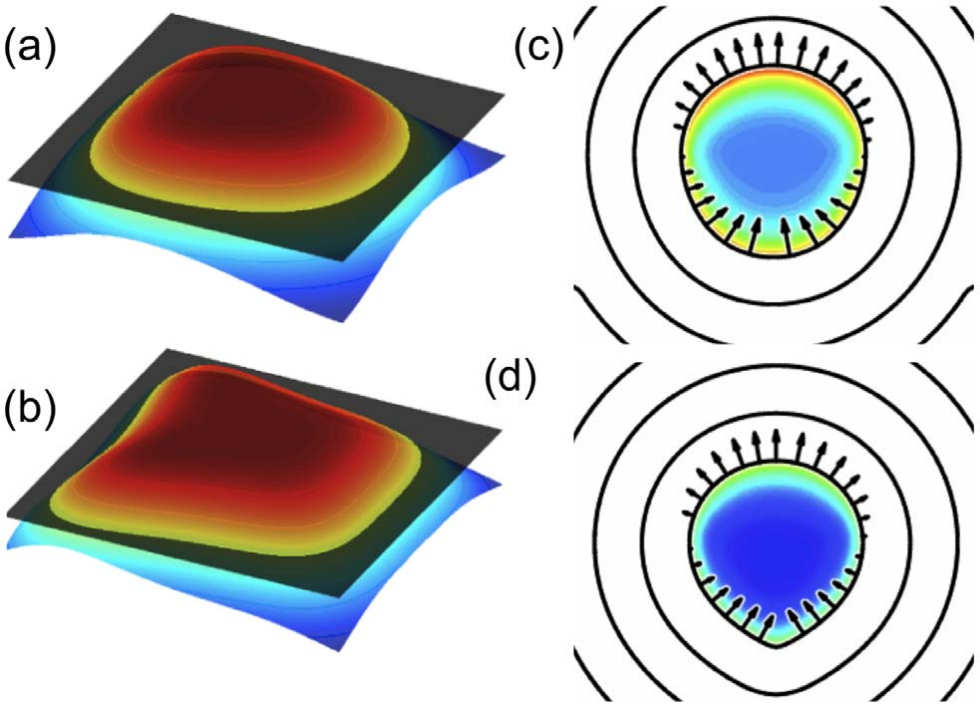
LSM. (a and b) Examples of the level set formalism, showing a characteristic level set function *φ*(*x*,*y*,*t*) at two time points. The level set (intersection of surface with the *φ* = 0 plane) represents the boundary of the cell at the given time points. (c and d) Two successive time points in a simulation of a crawling nematode sperm, analogous to Figure 7 from [48]. Curves are several level sets (contours) of the distance function, and arrows represent the velocity of the boundary. Original figure (panels c and d) was kindly supplied by Charles Wolgemuth and Mark Zajac.

In the LSM, pioneered by Osher and Sethian [47], there are no explicitly tracked material points on the cell edge. Instead, the boundary is considered to be a level set of a function describing the distance of any point in the 2-D domain from the boundary (see Figure 3). As a matter of convention, points inside the boundary are assigned a positive distance and points outside a negative distance value. The motion of this level set is described by an auxiliary “level set equation,” which incorporates a velocity determined by the problem. The benefits of this method are its ability to handle highly irregular shape changes with ease and to naturally incorporate boundary velocities in the level set equation. The downside of this method is that maintaining and propagating a high-quality level set function can be difficult. See [48] for further discussion and solutions to these issues.

#### Phase field method (PFM)

The PFM is different from the previous methods in that it is a diffuse interface method [49]. Here, a complex geometry is embedded into a larger regular domain such as a rectangle. A “phase field” (*φ*) is then defined to take distinct constant values inside (*φ* = 1) and outside (*φ = 0*) of the embedded domain. This phase field is smoothed so that the boundary is smeared over a (user determined) width *ε*. Thus, the boundary is represented by a region where ∇*φ* is large. The PFM does not track individual boundary points. Instead, boundary mechanics are computed based on the phase field itself.

In this framework, the PDEs are augmented to incorporate terms that account for the original boundary conditions. (A similar idea appears in the immersed boundary method.) If the domain is static, it is straightforward to solve the RD equations (see [50], [51]). For moving boundary problems, an evolution equation for the phase field *φ* is prescribed. This evolution equation can include a standard advective term to represent internal flows as well as a variation component determined by the variational derivative of a free energy functional with respect to *φ*. This free energy functional can encode curvature and other interface stresses that mainly affect the interface region where ∇*φ* is large. See [11], [52] for a brief description of this type of moving boundary calculation. A primary benefit of this method is that the problem is redefined on a rectangular geometry, which eliminates issues associated with boundary conditions on an irregular boundary. A substantial difficulty is the inclusion of terms such as ∇*φ* that are *O*(1/*ε*) in the augmented PDEs. This requires small mesh sizes in both time and space, increasing computational times.

#### CPM and/or Metropolis based simulations

The CPM is also known as the GGM. This method implements a Metropolis algorithm or its variants to control the expansion or retraction of the edge of the cell domain. In its basic form, that algorithm consists of the following steps: (1) A randomly chosen “cell edge” site is selected to either protrude outwards into a neighbouring empty site or retract inwards, leaving an empty site. (2) A term that corresponds roughly to an “energy” (the Hamiltonian, *H*) is calculated, based on an average over the entire cell edge and compared with that energy before the random move. The difference Δ*H* is then considered. (3) Whenever the “energy” is found to decrease (Δ*H*<0), the move is accepted and the calculation continues to another randomly selected site. In the opposite case (Δ*H*>0), the move is accepted with probability exp(−Δ*H*/*k_B_T*) where *k_B_* is Boltzman's constant and *T* the effective “temperature,” a parameter that governs fluctuations of the edge.

The Hamiltonian *H* is composed of a variety of terms specific to the problem under investigation. A typical example, from [12], is:
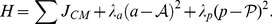
(3)Here the constant *J_CM_* is an energy per boundary site, 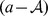 the deviation of the cell area *a* from a target area 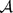, and similarly 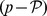 the perimeter deviation from some target length, a term describing an interfacial tension. Parameters such as *λ_a_* and *λ_p_* depict the resistance to stretching the cell or its perimeter.

The resulting simulations have an inherent stochastic character, leading to a fluctuating cell edge and abstract membrane ruffling. In general, the method does not aim to represent the complex physics of membrane fluctuations, being more concerned with motility of the cell as a whole. However, cytoskeletal fluctuations can be depicted and linked empirically to the actin polymerization ratchet mechanism as described in [12]. RD equations can be solved on the interior sites, though no fluid flows nor internal stresses or viscoelastic properties are then represented. The method makes for easier and faster computations for internal RD processes, allowing for more constituents to be simulated.

### PDE Integration/Solution Methods

**Figure 4:**
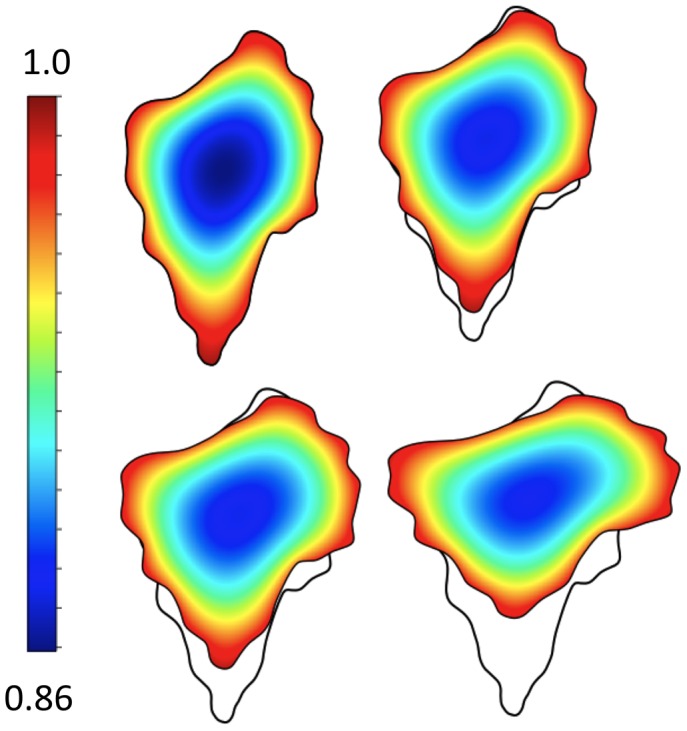
Finite volume 2-D simulations. Concentration of active GTPase tracks the changing cell shape in this simulation by Strychalski et al. [27]. The cut cell finite volume method is used together with the LSM. The velocity field is user-prescribed. RD equations for the GTPases are solved on the deforming domain, with particularly good performance for mass conservation (a technical issue plaguing many free boundary RD computations). Original figure kindly provided by Wanda Strychalski.

**Figure 5:**
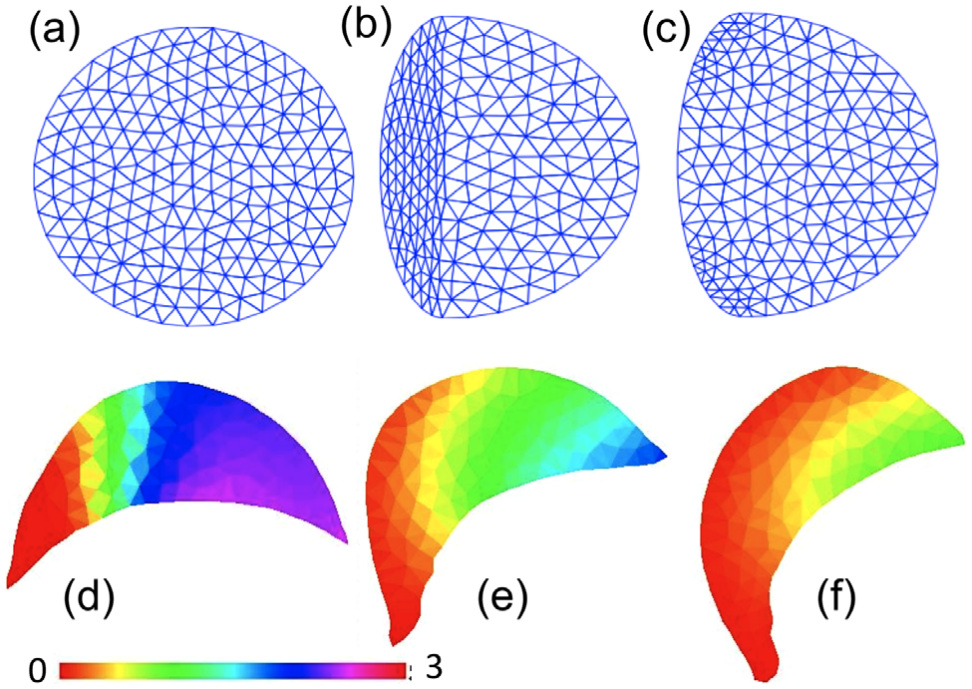
Finite element 2-D simulations. (a–c) An illustration of remeshing in a FEM computation. An initial domain (a) is subdivided into a triangular mesh, but as the domain deforms, the mesh becomes low quality (leading to poor accuracy) and too fine (increasing computational costs) in some parts. (b). The remeshing (c) is used to create a better and more regular mesh. (d–f) FEM simulations by Rubinstein et al. [7]. (Left to Right) Snapshots at three successive times, showing the free G-actin in a 2-D keratocyte turning simulation analogous to Figure 10 in [7]. The simulation depicts an experiment in which caged thymosin is photoactivated in the left part of the cell, causing G-actin depletion and cell turning. Colour bar: concentration of profilin-bound G-actin. Original figure (panels d–f) kindly provided by Boris Rubinstein.

The above methods describe ways to pose the moving boundary problem in mathematical language. Once this is accomplished, it is necessary to solve the PDEs describing reacting/diffusing/advecting quantities along with any auxilary equations describing boundary motions. The three techniques used to discretize model equations (whether singly or in some combination) are (1) finite differences (FDM), (2) finite volumes (FVM), and (3) finite elements (FEM). Each has its advantages and disadvantages. While FDM is the simplest, it does not readily handle irregular shapes and fails to conserve material (e.g., 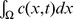, where *c*(*x*,*t*) is concentration of some conserved quantity) when the domain deforms. By comparison, FVM is a naturally conservative discretization method and more readily handles boundary conditions on irregular domains (see Figure 4 for an example). It is somewhat harder to implement than FDM and still has difficulties with irregular shapes. The FEM easily handles irregular shapes and boundary conditions but is by far the most complex of the three to implement. FEM also requires expensive remeshing with domain deformation to maintain a quality triangulation, as shown in Figure 5a–c. See Table 1 for the implementation method used in articles discussed here.

### 2-D Flattened Geometry Models

A diverse class of cell-shape simulation models fall into true 2-D computations. In contrast to the periodic 1-D domains described previously, such simulations describe thin cells or cell fragments where variations in the depth direction are negligible. Here, every point in the 2-D domain represents both dorsal and ventral cell membranes with the cytosol sandwiched in between. While physically separate compartments are not generally modelled, chemical species can be endowed with different properties (such as diffusivities) depending on whether they are membrane bound or cytosolic.

These models can be placed in several broad categories, as reviewed below. (1) Discrete cytoskeleton models: These models view the cytoskeleton as a (viscoelastic) network and describe it by a set of nodes connected by force elements such as springs and dashpots. (2) Continuum mechanical cytoskeleton models: These describe the cytoskeleton with continuous fields satisfying mass and momentum conservation. Forces such as adhesion and contraction are included via a stress tensor in the momentum equations. (3) Signalling models: Here the major concern is with hypothesized or biological regulators of the cytoskeleton and the RD equations they satisfy. As in previous examples, these tend to pay less attention to the cytoskeleton, linking signals to forces directly. (4) Hybrid models that consist of several aspects of the above.

### Actin Cytoskeleton in a Static Cell Shape

As noted above, the steady-state shape of keratocytes lends itself particularly well to investigations of actin growth, disassembly, and recycling in a fixed domain with varying degrees of detail of the biochemistry. While early models [14] considered the distributions of G- and F-actin, profilin, cofilin, thymosin, Arp2/3, and/or similar actin-associated proteins versus distance from the leading edge of the cell (in 1-D), more advanced computations have enabled similar investigations in 2-D and 3-D. Simulation platforms such as Virtual Cell (VC) can handle RD computations in irregular domains. We review this work in a concluding section on 3-D simulations.

A notable simulation employing static cell shape appears in [36] for the crawling speed of a nematode sperm cell. In this model-experiment work, realistic shapes of cells were used as 2-D computational domains, and results of the simulations were validated against experimental data. Assuming steady-state crawling of these cell shapes, the authors applied a multiphase fluid computation (reviewed below) to compute driving forces needed to produce observed cell speeds in cells of various sizes and shapes. One major finding is that such cells must have anisotropic MSP stiffness properties if model and data are to agree. Two further predictions were that larger cells crawl faster than smaller cells and that cells elongated along the axis of motion are faster than round cells.

A third computation on a static (keratocyte-shaped) cell domain is exemplified in Rubinstein and Mogilner [9]. Here the authors include viscoelastic actin mechanics, actin–myosin interactions with myosin flows, and cell-substrate adhesion. Model results for flow velocities and traction forces were compared with experimental data to show that the sweeping of myosin to the rear of the cell is important for the maintenance of the fan shape in motile keratocytes. In addition to 2-D fixed boundary simulations, several 1-D reductions of this model are proposed and analyzed (but one solution, for myosin distribution in the anterior-posterior axis is unfortunately incorrect), yielding analytical insights into the observed 2-D behaviour. The actin-myosin treatment here is more elaborate than that of [7]. (By comparison, the actin-myosin treatment is simpler in [11], [52].)

### Cytoskeletal Mechanics in a Deforming Cell Shape

Among the earliest examples of deforming cell calculations are those from the 1980s that treat cell sheets rather than individual cells. Oster and Odell [53], [54] and, later, Weliky and Oster [55], [56] used mechanically based spring-dashpot arrays to model a layer of epithelial cells. These were among the first simulations of their kind where the deformation of the domain was linked to specific mechanical forces. In [53], [54], for example, a simple bistable signalling system was invoked to regulate the rest length of a contractile element, thereby producing active contraction in response to a chemical or mechanical stimulus.

Bottino et al. [34] produced the first substantial 2-D simulation of a motile cell, based on the crawling motility of nematode sperm. It utilized a Lagrangian marker treatment of the boundary, tracking individual boundary nodes that were linked by Hookian springs (i.e., where force is proportional to deformation). The simulation incorporates a deforming lamellipodium attached to a nondeformable cell body. The essential result of this investigation is that bundling/swelling stresses of MSP can account for both protrusion and retraction. It is assumed that an imposed pH gradient affects MSP cross-linking. Near the front, strong cross-linking causes bundling and increases the persistence length of MSP polymers, creating a protrusive force. The bundling also stores energy. When the polymer gel is swept to the rear due to retrograde flow, cross-linking weakens, causing contraction and release of energy as the polymers contract to their shorter free persistence length. Computationally, the simulation is based on a collection of nodes linked by spring-dashpot branches representing bulk MSP properties with numerous rules controlling the behaviour of these nodes and force elements. The actual computation uses a set of Delaunay triangles (finite element method; FEM) and their associated Voronoi polygons. A 1-D reduction of this model was also analyzed to understand how the strain profile varies with respect to position in the lamellipodium.

In a paper with spirit similar to [34], Rubinstein et al. [7] presented a fully deforming 2-D moving keratocyte-shaped cell, as shown in Figure 5d–f. As with [34], this model utilizes a Lagrangian marker method with a finite element treatment of the advection-diffusion processes. Here the actin cytoskeleton and its properties are considered. The model is based on assuming that protrusion is normal to the leading edge, proportional to the local G-actin level and to a graded force-dependent factor. This is based on the GRE hypothesis. The authors assume uniform adhesion at the front and contraction due to myosin restricted to the 1-D rear edge. G-actin satisfies diffusion-transport in the free-boundary domain. F-actin is taken as a fixed graded density along the front edge, with constant depolymerization away from that edge that replenishes the G-actin pool. The cell domain is approximated as a thin sheet-like porous elastic solid with fluid flows satisfying D'Arcy's law [57]. G-actin pools in both polymerization-competent (profilin bound) and sequestered (thymosin bound) forms are tracked. An interesting finding is that local sequestration of G-actin by thymosin can give rise to turning behaviour, as observed experimentally (see simulation sequence in Figure 5d–f). The subsequent work by the same group [9], reviewed in a previous section, considers a fully viscoelastic actin network (but in a static domain).

Building on [36], Wolgemuth et al. [48] present a related model of nematode sperm crawling but in a level set computational framework (see Figure 3c–d) with additional biological features. As in [36], the cytoskeletal stress and cytosolic pressure produce force that powers forward pseudopod extension and cytoskeletal disassembly drives contraction. Cytosolic effects and MSP assembly are not explicitly modelled (rather, the MSP assembly is replaced by appropriate boundary conditions). Adhesion between the cell and substrate, incorporated as a resistive drag force, was assumed to differ sharply from front to back, with much lower adhesion values at the rear, where the cell body is located. By varying the point at which the transition occurs from high to low drag, the authors were able to obtain cell shapes that were either tear-drops or crescents. As in [36], an important feature of the model was the assumed anisotropic stiffness of the MSP polymer network, with higher front to back stiffness than side-to-side stiffness resulting from the observed anisotropic bundling structure of the MSP network.

Shao et al. [11], [52] developed a model similar to [9] using the PFM. They incorporate a viscoelastic treatment of the actin network, a drift-diffusion treatment of myosin that acts on that network, and discrete adhesion sites that can exist in either stick (spring force applied to the network) or slip (drag force applied to the network) states. The model includes effects due to tension and curvature of the cell edge (using phase field formalism to do so) and spontaneous polarization (by the wave pinning model [58], as in [13], [28]) in the actin kinetics. The authors concentrate on the effects of myosin contraction and adhesion strength. They track cell area and aspect ratio as a function of these strengths and verify experimental observations such as an inverse relationship between cell area and myosin activity.

Each of these investigations is aimed at understanding the balance between protrusion and contraction and how they give rise to shape change and motion. However, the biophysical elements incorporated vary greatly between one model and another. Bottino et al. [34] are primarily interested in the properties of the MSP polymer gel and how they give rise to both protrusive and contractile forces. The model in Wolgemuth et al. [48] focuses on how MSP gel anisotropy and graded adhesion affect cell shape. Rubinstein et al. [7] include both actin protrusion and myosin contraction but are more focused on biochemical details of actin recycling and G-actin sequestration. Shao et al. [11], [52] forgo such molecular details and instead concentrate on complex boundary mechanics and adhesion dynamics. A unifying model could be conceived with all or most of these elements. This would have been technically difficult to create de novo, and the role and importance of specific elements would have been obscured. Now that such separate components have been investigated on their own by various researchers, creating such unifying model to show how these modules interact becomes more tractable. For this reason, there is significant value in this diversity of investigations.

### The Evolving Cell as a Complex Fluid

The previous models characterize the cytoskeleton either using discrete viscoelastic spring/dashpot elements or with a continuum equation for F-actin. An alternative approach treats the cell interior as a complex multiphase fluid (see [59] for an excellent review). This approach was pioneered in a series of articles by Dembo, Alt, and coworkers [60]–[62]. Here, the cell interior is modelled as a two-phase interpenetrating flow, where the phases represent cytosol and cytoskeleton, each endowed with its own properties. The phases interact through drag and interconversion (representing production and degradation of the cytoskeleton). The cytosol and cytoskeleton are assumed to be viscous and visco-elastic, respectively, with hydrodynamic equations being used to encode their properties. This class of models is particularly suited for looking at the internal stresses in the cell and transmission of those stresses to the membrane.

In [32], this formalism was implemented with a finite element Lagrangian boundary method to investigate cytoskeletal force production in neutrophils. Two possibilities were considered. In the first, it was assumed that the cytoskeleton acts as an isotropic fluid where forces are based on hydrodynamic pressure. In the second, it was assumed that the cytoskeleton acts as an elastic scaffold capable of transmitting directed forces. In silico experiments were performed and compared with in vivo data. Experiments included neutrophil aspiration into a micropipette (negative pressure) as well as chemotaxis in a tube against an applied pressure (positive pressure). In the neutrophil chemotaxis experiment, the comparison of model and data for cell velocity as a function of applied pressure showed that the cytoskeleton behaves like a fluid rather than an elastic structure.

In [63], the same two phase fluid platform was applied to exploring dynamics, interactions, and feedback between cytoskeleton and adhesion in keratinocytes. Here it was assumed that adhesions influence the cytoskeleton through a drag force and that rupture of adhesions takes place due to cytoskeletal stresses. Similar to [33], this work was directed at understanding dynamic properties of cell shape. The authors showed that ruffling and periodic cycles of protrusion and retraction can result solely from a feedback loop between adhesion and cytoskeletal dynamics. A related article [10] is described in more detail in the section on 3-D models.

### Intracellular Signalling and Cell Shape Models

A few recent articles have concentrated on signalling dynamics in deforming 2-D domains. To depict the polarization of a cell, one or another set of RD equations is used to set up the initial intracellular chemical polarization that determines the front and back. One example is the simple two-component RD system based on GTPase signalling [58]. This tracks the concentration of a single GTPase in one of two states, an active (membrane-bound) and inactive (cytosolic) form, and polarizes by a “wave-pinning” mechanism. This RD system model can represent signal-induced transition to a robust persistent polarization. Hence, 2-D cell motility simulations based on it will generally result in a stable shape and persistently moving model cell.

The three articles [11], [13], [28] all similarly implement the same signalling model [58] in one or another way, but with distinct computational platforms. Vanderlei et al. [28] used Lagrangian immersed boundary formulation for a mechanical treatment of the cell edge, with a purely viscous cytosol. Wolgemuth et al. [13] used the LSM with a D'arcy flow representing the cytosol, and Shao et al. [11] include other cytoskeletal components with a more complex treatment of acto-myosin dynamics and cell edge mechanics. Cell shapes obtained in [13], [28] differ, being more tear-drop shaped in [28] and more oval in [13] due to a simple difference in the link assumed between active signal level and force on the cell edge. In [28], subthreshold signalling level was assumed to create active contraction (forces towards the cell interior), whereas in [28], only absence of an active force was assumed in such a case.

Other models with a significant signalling dynamics focus include [8], [12], [27], [64]. In [27], a finite volume-based LSM is used to investigate the effect of geometry changes on a GTPase like signalling model (Figure 4). Here, however, cell shape changes are user-prescribed rather than driven by internal chemistry. As the others [8], [12], [64] also include cytoskeletal aspects, we have classified them as hybrid models and these are discussed in the next section.

### Hybrid Models

Up to this point, we have mainly described models based on either cytoskeletal or signalling dynamics but not both. Here we discuss in more detail several recent models that include both aspects in some form. In [13] the LSM was applied to dissecting four possible models for 2-D motility of keratocyte-shaped cells. Cell shape was used as a readout. The conservation of chemicals and preservation of the cell's target area were given special consideration. The first model is based on G-actin diffusion and its polymerization into F-actin. The authors start with an initially polarized cell and assume that actin is polymerized close to the leading edge and depolymerized in a small elliptical region (defined ad hoc) near the rear. Cell area expansion is prevented by an effective restoring force.

**Figure 6:**
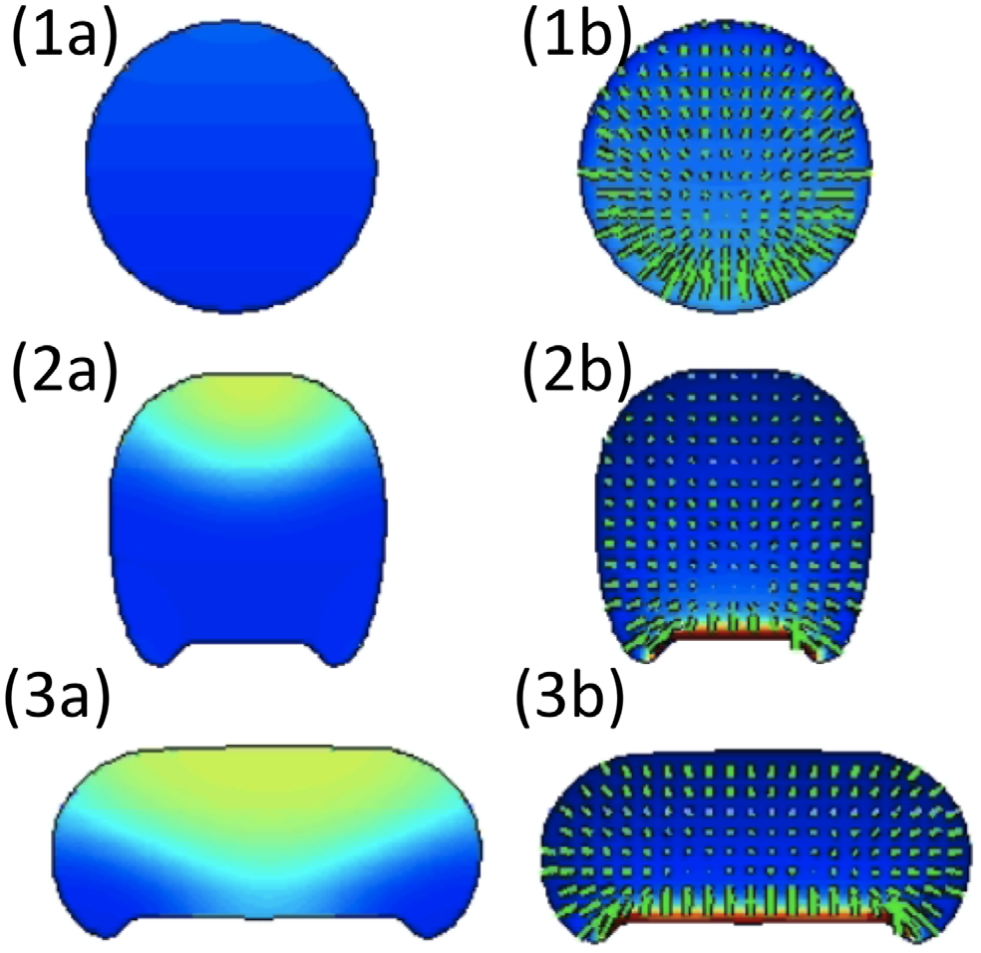
Keratocyte motility by LSM. The polarization and motility of a keratocyte-shaped cell similar to Figure 4 (E–F) and Movie S10, both in [13]. This model includes actomyosin contraction as well as the two-variable GTPase signaling module [58]. Bright colour indicates a high level of the signalling protein (left) and bound myosin (right), and the arrows indicate actin flow. Original figure kindly supplied by Charles Wolgemuth and Mark Zajac.

In their second model, the authors consider MT-directed vesicle traffic that supplies membrane to the expanding cell edge. They assume that rate of edge protrusion is proportional to the flux of vesicles for which the local MT tip density is a proxy. The MT organizing center (MTOC) from which MTs emanate as well as the MT parameters determine the polarity and motion of the cell. The third model, purely based on signalling, has been described and compared with analogous efforts [11], [28] in the previous section. In their final model, the authors consider acto-myosin as a viscous fluid with internal stress due to myosin (see Figure 6). Myosin exists in bound and unbound forms (with the former exerting forces). Bound myosin convects with the cell velocity, and its total is conserved in the cell. The cell velocity stems from the protrusion due to actin polymerization and the inward flow of actin due to myosin contraction. As an intermediate step, forces are considered for the myosin contraction model, and then converted to a velocity formulation. (A force velocity relation is not actually used.) The model has similarities to that of [9], but with a deformable cell edge. Interestingly, the first three models could, on their own, account for stable cell shapes, while the last, on its own, fails to do so. Coupled to the actin treadmill or the GTPase polarization model, the latter can replicate the stable and robust keratocyte motility phenotype. This is one of very few articles that uses a common platform to compare the performance of several biophysical models with important conclusions.

Two recent models in the literature [8], [12] have been assembled with more detailed and biologically explicit signalling dynamics. In [8], simulated keratocyte shapes were produced by coupling Arp2/3 activation to the activities of diffusing small GTPases, Cdc42, and Rac, interacting mutually and with Rho. Rho was used as a proxy for myosin activation and contractility. The growth and orientation of F-actin, the density of actin barbed ends, and those that impinge on the edge to cause protrusion forces were tracked. It was shown that this system would polarize to a small but finite transient-graded stimulus in the Cdc42 activation rate, and that the model cell would initiate and maintain polarization, while remaining sensitive to new stimuli.

**Figure 7:**
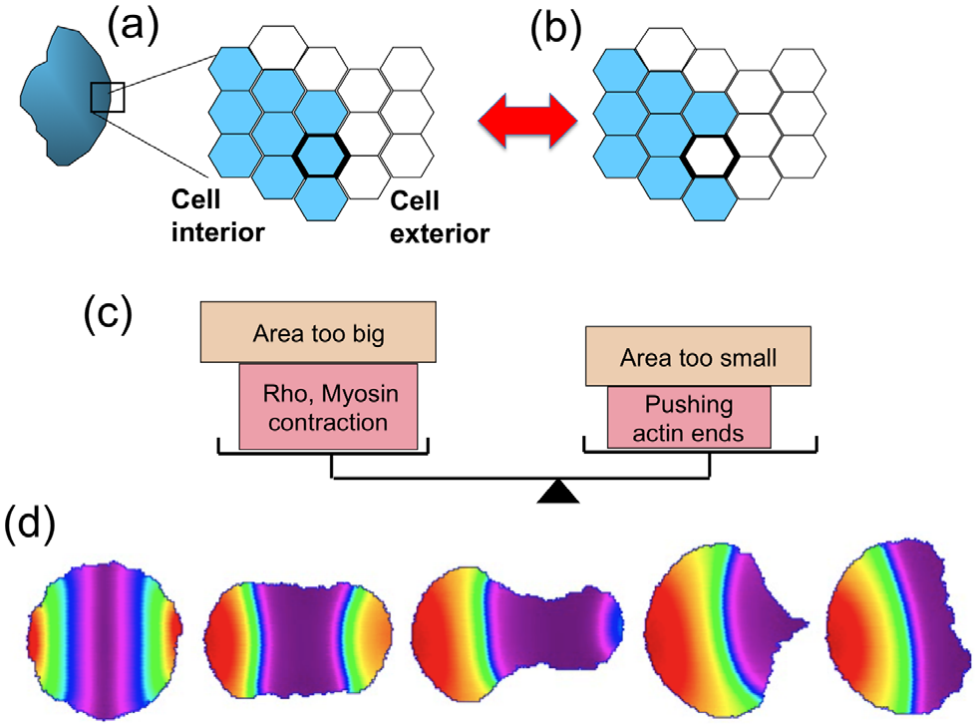
CPM simulations. In computations based on the CPM, a 2-D cell shape (a) is represented by a planar grid (b) using rectangular or, in this case, hexagonal elements that have two states: “inside” and “outside” the cell (blue and white, respectively). A Hamiltonian-based calculation (caricature in c) is used to determine if any of the edge sites change state. The transition shown in (b) depicts a retraction event where one cell interior site turns into an exterior site. This is favoured if the cell area is too large or if there is local contraction (presence of myosin or Rho GTPase). In [8], [12], each hexagonal site stores densities of actin filaments and barbed ends at six orientations. Also computed are the GTPases Cdc42, Rac, Rho, Arp2/3, and in [12] the phosphoinositides PIP_1_, PIP_2_, PIP_3_. In (d), a typical two-stimulus experiment is shown as a time sequence from left to right, as in Figure 6b of [12]. The appropriate level of feedback from PIP_3_ to Cdc42 and Rac makes for a smooth transition to a single “front.”.

Both [8], [12] use the CPM as a computational platform (see Figure 7). The effect of internal forces due to the cytoskeleton is incorporated by adjusting the Hamiltonian to include the pushing of actin filament barbed ends *P* at the edge, or the contraction due to local myosin activity *M*. Thus, the modified Hamiltonian was defined as 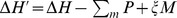 when the cell extends and 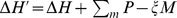 when the cell retracts. In this way, the presence of pushing forces biases against contraction, whereas the presence of myosin biases against protrusion. In both articles, the local activity of the GTPase Rho (*ρ*) above some threshold was used as a stand-in for myosin activity; i.e., *M*≈(*ρ*−*ρ_th_*).

In [12], a new layer of signalling, comprised of PIP_2_ and PIP_3_ (and their crosstalk with small GTPases), was added. One specific focus of that work was on how feedback from PIP_3_ to Cdc42 and Rac affects the cell's responses. When this feedback is absent, cells exposed to two opposing localized stimuli (e.g., at opposite poles) tend to produce two “fronts” that move in opposite directions. The cell takes on a dumb-bell shape, leading to significant deformation to the point of fission. Appropriate feedback from PIP_3_ leads to a “winner take all” decision, where one of the two fronts overtakes and dominates the other (Figure 7d). Such fine tuning also helps cells encountering an obstacle, so that a determination of direction of motility can be rapidly made. Too much feedback, it was shown, is also bad, as this tends to produce such high localized GTPase activity that the cell tends to freeze against an obstacle, rather than being able to navigate around it.

A second notable feature of [12] was the interplay between cell shape and internal signalling, a relatively less explored topic. Those articles that have explored such links [65]–[69] have predominantly focused on how the changing length, width, or height of a deforming cell results in better gradient sensing or in effective dilution or concentration of receptors or active signalling molecules. But the idea that regions of high curvature can accelerate the internal RD dynamics was shown in [12] to have significant impact on the speed of reorientation of a cell to new gradient directions.

In many previous models, signalling chemistry is upstream of (and unaffected by) cytoskeletal reorganization. Doubrovinski et al. [64] propose an alternative setting where signalling and cytoskeletal dynamics mutually affect each other. They explore the formation of dynamic actin structures such as travelling waves and spots. Two modules, representing F-actin dynamics and nucleation promoting factors (NPFs), are considered. The NPFs are assumed to nucleate F-actin (from a fixed G-actin pool), which in turn inactivates NPFs, creating a negative feedback loop. The authors use a Lagrangian marker method with a potential based free energy formulation to account for transmission of internal forces to protrusion when such transient actin structures interact with the cell edge. They show that a Turing instability of the NPF equations spawns a variety of spatio-temporal F-actin patterns that lead to interesting cell behaviours such as directed motion, breathers (rhythmic cycles of expansion and contraction of the cell area), and rotating protrusions.

## Cell Motility and Shape in 3-D Computations

### Cytoskeleton Assembly in Static Cell Shape

Extensions of 1-D models for actin assembly in [14] to static 3-D domains with keratocyte shape have been performed in [70]. This investigation uses the freely available VC environment [71] capable of simulating reaction/drift/diffusion equations on arbitrarily shaped but (so far) static domains in 2-D and 3-D. The model consists of diffusion-drift-reaction equations that describe G-actin cycling with spatially distributed assembly and disassembly of F-actin. This platform was used specifically to address questions about the effect of local versus global F-actin turnover.

In [72], an even larger and more biochemically detailed VC simulation includes numerous nucleotide states for actin monomers and actin binding proteins such as profilin, thymosin, cofilin, and capping protein. Arp2/3 is activated by membrane-bound N-WASP, and the polymerized actin experiences retrograde flow due to an imposed internal velocity field. Kinetic parameters are based on biological literature, and the steady-state distribution of all substances is visualized in a 3-D cell-shaped domain. One finding that emerges from this model is existence of a sharp transition layer between the zone where actin assembles and disassembles. Another finding is that cofilin can act as both enhancer and inhibitor of actin polymerization, depending on the level of capping protein. Finally, a sensitivity to Arp2/3 debranching emerges as one key parameter.

The model in [72] consists of around 50 PDEs and another 10 or so algebraic equations for biochemistry on its own, without mechanics nor viscoelastic behaviour. This means that the simulation requires tens of days of computation time per run (but smaller 2-D versions are faster). One of the attractive features of VC is that it is amenable to experimentation by non-mathematicians. Thus, such open models may allow for translatability of the research from modellers to biological researchers. One difficulty, however, is that the assembly of such large models entails combinatorial points of decisions about implementation details. These are hard to describe in full detail, let alone change or manipulate. Thus, to some extent, the platform has aspects of a black box that may be challenging to comprehend by outside researchers.

Hybrid models of these kinds have seen increased interest recently, as researchers have moved from investigating the functions of individual elements underlying motility to interactions between these elements. The increased complexity of these models comes with increased diversity owing to the combinatorial ways that various biological aspects can be included and computational methods used. Further development of these hybrid platforms will need to be undertaken to begin to investigate more elaborate cellular functions, such as the ability to navigate in complex environments and to resolve multiple conflicting stimuli.

### Deforming 3-D Cell Shape with Internal Cytosol and Cytoskeleton

**Figure 8:**
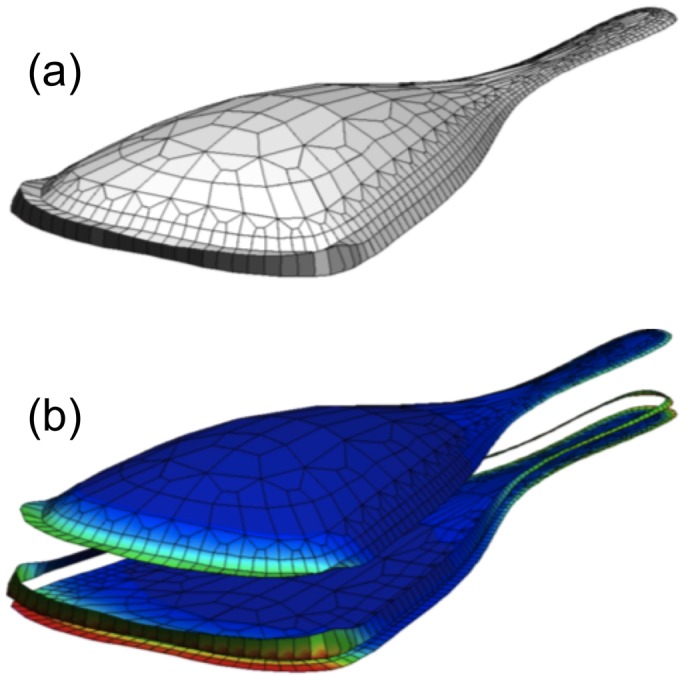
A 3-D simulation. A simulation of a fibroblast cell shape in 3-D analogous to that of [10]. Two-phase fluid modeling of a fibroblast-like cell with Cytopede [73]. (a) Rendering of the cell surface. (b) Exploded surfaces with coloring by level of cytoskeletal density. Original figure kindly produced and provided by Marc Herant and Micah Dembo.

A pioneering article for fully deforming 3-D free boundary problem for cell shape in cytokinesis was [61], based on this idea of multiphase fluids. One of the recent remarkable contributions to the field has been the deformable 3-D simulations of [10], [73] (see Figure 8). These articles are based on low-Reynolds number hydrodynamic finite element simulations of cytoskeletal rheology and protrusive activity leading to 3-D cell shapes (Cytopede) [10], [73]. The model assumes a thin (but dynamically changing) cell geometry. A highly specialized finite element procedure is employed where the cell is endowed with an anisotropic mesh that treats the cell depth differently than the planar direction parallel to the substrate. Included in the model are the two-phase fluid equations (mass and momentum balance) together with constitutive equations (for network polymerization), vertical contractile forces (to keep the lamellipod flat), and boundary conditions (to depict actin nucleation factors at a leading edge). A single diffusible “messenger” is activated along some fraction of the leading edge to nucleate actin polymerization. Stress terms are used to represent protrusion of the front (as a cytoskeleton-membrane repulsion term) and adhesion to the substrate. The cytoskeleton is assumed to stick to the ventral (substratum) cell surface, whereas surface tension controls the top (free) cell surface.

The output reveals detailed cytoskeletal velocity fields as well as cytoskeletal volume fractions over the cell as it polarizes and deforms (starting from a disk-shaped resting cell). Remarkably, this article shows that fibroblast and keratocyte morphology and motion can be achieved by modifying two parameters: the fraction of the cell perimeter at which actin is polymerized (50% in keratocytes versus 25% in fibroblasts) and adhesion at the rear of the cell, which is weaker in keratocytes.

## Influence of Modelling Efforts

Although it may be difficult to pinpoint direct connections between specific simulations and experimental tests, the modelling efforts reviewed here have influenced the exploration and understanding of underlying biological mechanisms. Perhaps the clearest example of synergistic cycles of modelling and experiment is the work of Mogilner, Theriot, and coworkers on keratocyte shape/motion. Spawned by the seminal article by Grimm et al. [6] and carried on in [40], [74], for example, this investigation demonstrates a clear collaborative path incorporating theory and experiment that has spanned nearly a decade.

Other examples are more abstract. Rubinstein et al. [7], [9] suggested that cytosolic flows are important for motility. This work motivated the quantification of the fluid flows in keratocytes, as described, for example, in [75]. Prass et al. [76] also took on the challenge of directly measuring forces of protrusion in keratocytes. The simulation studies of Herant and Dembo on neutrophil aspiration and phagocytosis were influential in bioengineering works such as [77], [78]. The original simulation of the nematode sperm motility [34] was followed up by the experimental-theoretical work in [35]. Investigation of the roles of GTPases described in [8] led to links with analogous proteins important in plant development (ROPs) [79] and cell division (PARs) [80].

## Discussion

As evident from this survey, simulations of cell motility have a wide-ranging set of goals, from conceptual proof of concept to detailed biological comparisons. Two sources of complexity arise when approaching this problem, namely biological complexity and computational complexity. Our survey of the literature has been organized along these two themes, as captured in Figure 1. Other reviews [59], [81], [82] concentrate on different but complementary aspects of motility modelling (from single actin filaments to whole cell models).

Models that simplify the biological details, or consider a 1-D spatial setting, can in some case be analytically treated and generally lead to simpler computations. This leads to more complete parameter studies and understanding of model properties. However, these models may be difficult to link to the biology and generally provide only qualitative agreement with observations. Such 1-D models and models with fewer components provide good stepping stones for more detailed and complete treatments. Building in successive layers of detail gradually allows the researcher to understand the effect and role of each piece more thoroughly. This helps to build an understanding that is rarely possible when a highly complex model is the starting point of an investigation. That said, this progression does not necessarily lead to a hierarchy of models per se, since simple models have many distinct starting points and hypotheses. As evident from this review, there are many approaches, and they all bring something to the table.

As we have noted, a number of sources of computational complexity arise in the development of 2-D simulations, even of the biologically simple models. One source is the inevitable challenge of the free boundary problem linked to deforming cell simulations. In the case of 1-D perimeter models, formal analysis can be done in some cases, but the free boundary problem still provides technical challenges. The technical challenges and shear computational costs increase dramatically when moving to 2-D (and even more so subsequently in 3-D). As outlined, a number of platforms are currently being considered for dealing with these challenges. Examples include force-based models (e.g., [17], [18]) or energy-based models [8], [12]. This issue is somewhat independent of the biological content of the model.

Attempting to include more biological detail also presents computational challenges. The issues, as we have discussed, reside both in the calculation of RD systems or viscoelastic fluid flows in deforming domains, as well as treatment of the boundary itself. A detailed model for signalling pathways results in a large system of RD PDEs. These models tend to have large numbers of parameters, only some of which are constrained by data. Similarly, behaviour of these models can be sensitive to particular mathematical assumptions that make it challenging to map interesting parameter regimes using traditional techniques. (See, however, a new analysis method [69] that provides an efficient tool for this purpose in cases where there is a large membrane/cytosol diffusion disparity.) The treatment of viscoelastic flows is a bit more standard given the huge computational fluids literature. Treatment of boundary mechanics has proven to be more problematic and is a source of differences among models. Due to these complexities, all methods devised to date take certain shortcuts on some aspects, while handling other aspects effectively. This has resulted in a wide diversity in the modelling literature. Many computations impose material conservation correction [48] on an otherwise nonconservative computational scheme. Others impose an area constraint in lieu of describing membrane tension [52].

What emerges from this area of research so far? One universal theme is that there are many ways of accounting for keratocyte shapes and steady motion. Very diverse models with distinct components and simulation platforms have reproduced this kind of cell shape and gliding movement pattern. A second is that any number of simple signalling systems can mimic cell polarization and active patches on cell perimeters. A third is obvious disagreement between practitioners as to what are the fundamental activators/inhibitors (or similar signalling components) that control amoeboid motility (in the favorite organism, *Dictyostelium*) and whether they initiate pseudopods or affect the pseudopod competition cycle. A fourth theme, and second example of diverging views is the basic view of a cell and its cytoskeleton, whether as interpenetrating fluids, viscoelastic material, collection of discrete filaments and linkers, or some other entity. This greatly colours the approaches, the types of simulations, as well as the questions addressed.

What are other issues and challenges we face in carrying out this kind of research? One problem, as hinted above, is that we are still far from understanding how to best describe the “material” that cells are made of. Even if we could compute in detail the spatio-temporal distribution of the cytoskeleton, its regulators, and motors that act on it to create stresses, we would still be far from answering this question fully. We still do not have adequate ways of linking the density and structure of the cytoskeleton to macroscopic parameters such as viscosity, elasticity, and internal stress distributions. A more practical challenge is the high entry fee to this realm of research. At the moment, every group has to build complex software from scratch leading to fragmentation and difficulty comparing results. Further, the skills needed to develop simulation software are largely disjointed from those needed for model development and biological exploration. The VC project [71] has mitigated this in the static cell modelling world, but no such project is available for dynamic cell modelling so far.

Moving forward in the face of such complexities, there are a number of ways models have and can continue to advance understanding. (1) Models that maintain a close link to biology via experiments have helped to bootstrap advanced experimental techniques. Especially notable are contributions with cycles of model and experiment that complement one another. (2) Models that can rule out biological hypotheses on the basis of physical principles or predictions are also extremely helpful, as they provide definitive conclusions. (3) Failing such direct links to data, models that gradually build complexity, illustrating what new behaviour stems from additional detail are particularly helpful in gradually building a mechanistic understanding of the processes that underlie motility.

As far as future challenges, we point to one obvious gap. There has been no computation so far for cell shape changes during motion on a 3-D collagen network or navigating in 3-D. There is much new evidence that this type of cell motility differs substantially from the flattened motility seen in vitro. This remains a challenging visualization and computational problem for the future. Models have already been used as experimental tools, in aid of answering clearcut biological questions (e.g., “Is it reasonable to consider VASP as an anti-capping agent?” [40]). In the next years, closer model–experiment links will be important to move this field forward. We need experiments to help narrow the focus on competing mechanisms, to provide narrower parameter regimes, and to test and validate models so that we can gradually build more detailed understanding based on solid facts. We need models to suggest conceptual frameworks, to organize disparate biological findings, and to provide a rigorous platform for testing ideas.
